# Statement of Retraction: Exosome-delivered BMP-2 and polyaspartic acid promotes tendon bone healing in rotator cuff tear via Smad/RUNX2 signaling pathway

**DOI:** 10.1080/21655979.2024.2299604

**Published:** 2024-02-20

We, the Editors and Publisher of the journal *Bioengineered*, have retracted the following article:

Lei Han, Hong Liu, Huajun Fu, Yugen Hu, Weili Fang & Junsheng Liu (2022) Exosome-delivered BMP-2 and polyaspartic acid promotes tendon bone healing in rotator cuff tear via Smad/RUNX2 signaling pathway, Bioengineered, 13:1, 1459-1475, DOI: 10.1080/21655979.2021.2019871

Since publication, significant concerns have been raised about the integrity of the data and reported results in the article. When approached for an explanation, the authors did not provide their original data or any necessary supporting information. As verifying the validity of published work is core to the integrity of the scholarly record, we are therefore retracting the article. All authors listed in this publication have been informed.

We have been informed in our decision-making by our editorial policies and the COPE guidelines.

The retracted article will remain online to maintain the scholarly record, but it will be digitally watermarked on each page as ‘Retracted’.

